# Analysis of Genetic Regions Related to Field Grain Number per Spike From Chinese Wheat Founder Parent Linfen 5064

**DOI:** 10.3389/fpls.2021.808136

**Published:** 2022-01-05

**Authors:** Ling Qiao, Hanlin Li, Jie Wang, Jiajia Zhao, Xingwei Zheng, Bangbang Wu, Weijun Du, Juanling Wang, Jun Zheng

**Affiliations:** ^1^College of Agronomy, State Key Laboratory of Sustainable Dryland Agriculture (in Preparation), Shanxi Agricultural University, Jinzhong, China; ^2^Institute of Wheat Research, Shanxi Agricultural University, Linfen, China

**Keywords:** founder parent, Linfen 5064, wheat, grain number per spike, quantitative trait locus

## Abstract

Wheat founder parents have been important in the development of new wheat cultivars. Understanding the effects of specific genome regions on yield-related traits in founder variety derivatives can enable more efficient use of these genetic resources through molecular breeding. In this study, the genetic regions related to field grain number per spike (GNS) from the founder parent Linfen 5064 were analyzed using a doubled haploid (DH) population developed from a cross between Linfen 5064 and Nongda 3338. Quantitative trait loci (QTL) for five spike-related traits over nine experimental locations/years were identified, namely, total spikelet number per spike (TSS), base sterile spikelet number per spike (BSSS), top sterile spikelet number per spike (TSSS), fertile spikelet number per spike (FSS), and GNS. A total of 13 stable QTL explaining 3.91–19.51% of the phenotypic variation were found. The effect of six of these QTL, *Qtss.saw-2B.1, Qtss.saw-2B.2, Qtss.saw-3B, Qfss.saw-2B.2, Qbsss.saw-5A.1*, and *Qgns.saw-1A*, were verified by another DH population (Linfen 5064/Jinmai 47), which showed extreme significance (*P* < 0.05) in more than three environments. No homologs of reported grain number-related from grass species were found in the physical regions of *Qtss.saw-2B.1* and *Qtss.saw-3B*, that indicating both of them are novel QTL, or possess novel-related genes. The positive alleles of *Qtss.saw-2B.2* from Linfen 5064 have the larger effect on TSS (3.30%, 0.62) and have 66.89% in Chinese cultivars under long-term artificial selection. This study revealed three key regions for GNS in Linfen 5064 and provides insights into molecular marker-assisted breeding.

## Introduction

Founder parents are not only successful cultivars that are cultivated in large areas but are also used extensively as parents in breeding programs. These valuable genetic resources are crucial to Chinese wheat breeding programs (Zhuang, 2003). Analyzing the genetic diversity of founder parents and the genetic basis of their widespread success can provide a foundation for more efficient use of these germplasm resources.

A Chinese wheat founder parent named Linfen 5064 is the pedigree of more than 80 high-quality strong gluten cultivars in China. Linfen 5064 has the strong-gluten trait, a high grain number per spike (GNS), and excellent agronomic traits (Qiao et al., 2018). Linfen 5064 and cultivars derived from it not only have high yields but have also been used as the main parents for improving wheat quality in Chinese breeding programs. The use of Linfen 5064 as the founder parent addressed three difficult points in the breeding for strong-gluten wheat (Qiao et al., 2018). The first difficultly is that quality is negatively correlated with GNS and thousand kernel weight (TKW). Chinese wheat cultivars with premium grain quality, such as Xinong 20, Fengdecun 5, Shiluan 02-1 and Jimai 20, usually have lower GNS and lower yields. The GNS of Linfen 5064 and cultivars and lines derived from it have higher yields than other high-quality cultivars. The second difficult point is that dwarfism is associated with late maturity. Linfen 5064 does not show this association as it matures early and is a semi-dwarf height of about 75 cm. Finally, Linfen 5064 overcomes the need to have the glutenin subunit combination 5 + 10 for good quality, since it lacks these subunits yet still has good quality. Therefore, the utilization of valuable traits of Linfen 5064, and the successful future breeding program of Wheat, it is essential to explore and analyze their genetic base.

In most wheat cultivars, a spike usually generates more than 10–20 spikelets, and each spikelet can differentiate into 9–10 florets (Cui et al., 2008). The differentiation of bract and floret primordia determines the number of spikelets and initial florets. During floret development, 60–80% of the initial florets either abort or otherwise lose fertility (Guo et al., 2015). The number of surviving florets which can eventually develop into grains determines the number of grains per spike (Zhang et al., 2021). GNS shows high heritability (Isham et al., 2021). Increasing GNS is an important way to increase grain yield. GNS can be divided into total spikelet number per spike (TSS), fertile spikelet number per spike (FSS), base sterile spikelet number per spike (BSSS), top sterile spikelet number per spike (TSSS), and grains per spikelet. The heritability of TSS was higher (Isham et al., 2021), but the number of grains per spikelet and spikelet propagation ability were greatly affected by the environment. The map-based cloning of common wheat genes lags that of other crops because of wheat's large genome size. Consequently, most studies focus on the quantitative trait loci (QTL) level of analysis, especially genes/QTL that control yield traits.

Hundreds of QTL for GNS have been found to be distributed across the 21 wheat chromosomes (Börner et al., 2002; Huang et al., 2006; Narasimhamoorthy et al., 2006; Li et al., 2007; Ma et al., 2007; Wu et al., 2012; Jia et al., 2013; Zhang et al., 2016; Cui et al., 2017; Guan et al., 2018; Keeble-Gagnere et al., 2018; Onyemaobi et al., 2018; Su et al., 2018; Deng et al., 2019; Fan et al., 2019; Liu et al., 2019; Yao et al., 2019). Some genes related to GNS had been reported, such as homology-based cloned genes *TaTAR2.1-3A* (Shao et al., 2017), *TaCWI-4A* (Jiang et al., 2015), *TaMOC1-7A* (Zhang et al., 2015), *TaSnRK2.9-5A* (Rehman et al., 2019), *TaAPO-A1* (Muqaddasi et al., 2019), *TaGW8-B1* (Yan et al., 2019), *TaPHR3-A1* (Zheng et al., 2020), the *Q* gene (Chuck et al., 1998; Debernardi et al., 2017; Xie et al., 2018), and genes *GNI-A1* (Sakuma et al., 2019) and *WFZP* identified *via* map-based cloning (Du et al., 2021). Genes for other traits of agronomic importance, such as flowering time (FT) and plant height (PH), can have significant effects on grain yield (Cuthbert et al., 2008; Zhou et al., 2017; Guan et al., 2018). *Ppd-1* participates in the regulation of flower spike development in wheat, which affects the number of spikes and seed setting (Boden et al., 2015).

Although many QTL/genes associated with GNS have been reported in wheat, the major and stable QTL identified under multiple environments are still limited. In addition, the biparents used for mapping were mostly accessions aim at certain traits rather than founder cultivars, the use of QTL identified need long-term backcross process which is time-consuming and low efficiency. We especially used founder parent and core cultivars in breeding as biparents for mapping, the loci obtained and markers developed are easily used in breeding, also provide evidence on utilization of the derivatives. Two doubled haploid (DH) populations (Linfen 5064 × Nongda 3338 and Linfen 5064 × Jinmai 47) were analyzed for five GNS-related traits over the nine experimental locations/years to (1) identify and validate major, stable QTL for GNS that can be used for molecular marker-assisted breeding and (2) identify genetic regions associated with GNS of Linfen 5064, elucidate the genetic mechanism of GNS in the founder parent, and discover favorable allele variations.

## Materials and Methods

### Plant Materials

A total of two DH populations were used, 192 lines from the cross Linfen 5064 × Nongda 3338 (LN) and 194 lines from the cross Linfen 5064 × Jinmai 47 (LJ). Linfen 5064 is a Chinese wheat founder parent with strong gluten, a high GNS, and an excellent array of other characteristics (Qiao et al., 2018). Nongda 3338, developed by China Agricultural University, is a “core parental” breeding line for the North China Winter Wheat Breeding Program with high general combining ability and the dwarfing genes *Rht-B1b* and *Rht-D1b* (Kabir et al., 2015). Jinmai 47 has the advantages of drought tolerance, stable yield, and a high utilization rate of water and fertilizer (Song et al., 2017). The phenotypic difference between the two cultivars and Linfen 5064 was significant and there was obvious trait separation in the population. LN was used for QTL analysis and LJ was used to validate the effects of putative QTL identified in LN.

### Field Evaluation

The two DH populations were planted as a single replication in three locations in 2018–2019, 2019–2020, and 2020–2021. Locations were in the Yaodu district in Shanxi province of China, at Linfen (36°08′N, 111°52′E, altitude 450 m) (19 YD, 20 YD, and 21 YD), Hancun (36°25′N, 111°67′E, altitude 450 m) (19 HC, 20 HC, and 21 HC), and Yuncheng (35°15′ N, 110°98′ W, altitude 369 m) (19 YC, 20YC, and 21 YC). The seed was sown in two 1.5 m rows per line spaced 0.3 m apart at 21 seeds per row. Field management practices were those commonly used in wheat production in the region.

### Phenotypic Evaluation and Data Analysis

Ten days before harvest, data of five spike traits, TSS, BSSS, TSSS, FSS, and GNS, were collected by randomly choosing 10 plants in each line. FSS = TSS-BSSS-TSSS. The best linear unbiased prediction (BLUP) of target traits in different environments (Smith et al., 1998) and the broad-sense heritability (*H*^2^) were obtained using SAS (SAS Institute, Cary, NC, USA; https://www.sas.com). The SPSS18.0 software (SPSS, Chicago, Illinois, USA; http://en.wikipedia.org/wiki/SPSS) was used to perform Student's *t*-test (*p* < 0.05) and correlation analysis of phenotype values in different environments.

### Genetic Map Construction and Linkage Analysis

The two DH and parental lines were genotyped with a 15 K single-nucleotide polymorphism (SNP) panel developed based on 20 resequencing datasets, 1,520 genotyping datasets collected globally from multiple platforms, and publicly released resequencing and exon capture data. These datasets were developed and optimized using GenoBait technology to finally yield 14,868 mSNP regions for use in this study.

The genetic map of LN was constructed using IciMapping 4.1 (Meng et al., 2015) and JoinMap 4.0. Markers were binned if the correlation coefficient between them was 1 using the BIN function in IciMapping 4.1 according to the method reported by Winfield et al. (2016). WinQTLCart version 2.5 (Wang et al., 2012) for composite interval mapping was used to detect QTL. The minimal logarithm of odds (LOD) score to accept the presence of a QTL was set at 2.5. QTL was considered major when more than 10% of the phenotypic variation was explained in at least one environment and it was detected in at least three environments, including the BLUP dataset. QTL either <1 cM apart or sharing common flanking markers were treated as a single locus.

### Validation for the Major QTL Identified

Peak SNPs for stable QTL identified in the LN population were genotyped in the LJ population. The differences in spike-related traits between both groups in the LJ population were analyzed with a *t*-test in SAS V8.0.

### Genes Identified in the Major QTL

Genes within the target region of major QTL were obtained using the genome browser (JBrowse) on the WheatOmics-bata website http://wheatomics.sdau.edu.cn/ (Ma et al., 2021). Functional annotation and enrichment analysis of genes in these regions were done using the gene ontology (GO) database and the R package cluster Profiler. Analysis of orthologs between wheat and rice used the Triticeae-Gene Tribe website (http://wheat.cau.edu.cn/TGT/). The expVIP public database (http://www.wheat-expression.com/) was used to search for the expression data of genes in 16 tissues and organs, perform log2 conversion processing, and analyze the expression patterns of genes.

The R software package LD heatmap of major QTL was used to draw the linkage disequilibrium heatmap according to the resequencing data in 145 landmark cultivars that were downloaded from https://wheat.cau.edu.cn/WheatUnion/ (Hao et al., 2020).

## Results

### Phenotypic Variation and Correlations of Five Traits in Nine Environments

Linfen 5064 had lower values for TSS and TSSS, and a higher value of GNS than Nongda 3338 (Table 1). The spike traits of the DH population showed continuous variation, suggesting multigene genetic control. The estimated *H*^2^ of five traits ranged from 0.78 to 0.92, indicating that these traits were significantly affected by genetic factors (Table 1). The Pearson correlation coefficients among different environments were significant (*P* < 0.05, Supplementary Table S1). Better among-environment correlations were observed for TSS than for FSS, TSSS, BSSS, and GNS.

**Table 1:** Phenotypic variation and distribution of five spike-related traits in parents and the doubled haploid (Linfen 5064 × Nongda 3338) in nine field trials.

**Traits**	**Environment**	**LF 5064**	**ND 3338**	**MIN**	**MAX**	**Mean**	**SD**	***H*** **^2^**
TSS	19HC	16.47	16.82	13.20	21.60	17.09	1.56	0.90
	20HC	18.12	18.36	15.50	23.20	18.31	1.30	
	21HC	17.07	17.12	13.60	20.80	17.31	1.17	
	19YD	19.13^*^	21.07^*^	17.40	23.40	20.34	1.08	
	20YD	20.13	20.87	17.60	25.80	20.31	1.07	
	21YD	19.00^**^	20.60^**^	17.40	25.40	20.12	1.20	
	19YC	19.27^**^	21.65^**^	16.20	26.00	20.58	1.34	
	20YC	20.60	21.00	16.67	24.20	20.73	1.17	
	21YC	18.80^*^	20.73^*^	17.40	24.20	20.16	1.12	
	BLUP	18.83	19.74	17.80	23.12	19.44	0.85	
FSS	19HC	15.72	14.82	12.80	20.30	16.32	1.55	0.89
	20HC	17.12	16.83	14.00	23.20	17.28	1.31	
	21HC	16.60	16.99	12.20	20.20	16.99	1.17	
	19YD	17.20	16.54	9.60	20.20	17.39	1.43	
	20YD	19.40	19.13	16.20	24.40	18.85	1.16	
	21YD	17.73	18.33	15.20	22.80	18.39	1.32	
	19YC	17.87	19.23	13.80	22.20	18.29	1.35	
	20YC	18.53	17.40	14.67	22.00	18.26	1.25	
	21YC	16.40	17.13	13.60	21.80	17.54	1.41	
	BLUP	17.63	18.01	15.13	20.75	17.70	0.78	
TSSS	19HC	0.00	1.27	0.00	2.00	0.23	0.36	0.78
	20HC	0.44^*^	1.53^*^	0.00	4.60	0.44	0.55	
	21HC	0.13	0.00	0.00	2.20	0.09	0.24	
	19YD	0.13^**^	1.40^**^	0.00	4.40	0.81	0.95	
	20YD	0.20	0.53	0.00	2.00	0.28	0.39	
	21YD	0.33	0.33	0.00	4.00	0.51	0.63	
	19YC	0.20	1.83	0.00	3.80	0.78	0.75	
	20YC	0.67	2.27	0.00	4.40	0.76	0.73	
	21YC	0.20	1.53	0.00	6.40	0.85	1.02	
	BLUP	0.32	1.05	0.13	2.15	0.53	0.32	
BSSS	19HC	0.75	0.73	0.00	2.00	0.53	0.47	0.92
	20HC	0.56	0.00	0.00	2.00	0.59	0.50	
	21HC	0.33	0.13	0.00	1.40	0.22	0.29	
	19YD	1.80	3.13	0.60	4.40	2.14	0.81	
	20YD	0.53	1.20	0.00	2.80	1.18	0.56	
	21YD	0.93	1.93	0.00	3.00	1.21	0.58	
	19YC	1.20	0.58	0.00	3.20	1.51	0.57	
	20YC	1.40	1.33	0.20	3.60	1.71	0.63	
	21YC	2.20	2.07	0.00	3.60	1.78	0.67	
	BLUP	1.09	1.23	0.46	2.08	1.21	0.32	
GNS	19HC	53.80^*^	40.84^*^	26.00	66.80	47.51	7.34	0.81
	20HC	56.78	47.33	22.80	68.80	47.84	7.36	
	21HC	52.20	45.33	29.20	70.60	52.92	6.54	
	19YD	42.27	34.60	13.20	104.20	37.17	8.20	
	20YD	52.80	47.20	31.20	65.20	44.76	5.94	
	21YD	51.93	37.47	25.00	74.20	45.84	7.63	
	19YC	49.87	45.47	20.80	68.00	44.37	7.73	
	20YC	50.03	37.27	17.20	58.80	42.32	7.60	
	21YC	46.27	33.67	13.50	70.60	42.78	8.67	
	BLUP	49.64	42.69	32.39	54.31	45.04	3.87	

*H^2^, broad-sense heritability; BLUP, best linear unbiased prediction*.

* *Significant at p < 0.05*;

** *significant at p < 0.01*.

Phenotypic correlations among spike traits were evaluated using the BLUP dataset (Table 2). GNS significantly and positively correlated with FSS and TSS. GNS and FSS significantly and negatively correlated with BSSS and TSSS (*p* < 0.01, Table 2). The order of correlation coefficient with GNS were FSS (0.630) > TSSS (−0.437) > TSS (0.336) > BSSS (−0.162). These results showed that FSS and TSSS exerted great influence on GNS.

**Table 2:** Coefficients of pairwise Pearson correlations among five spike-related traits in the DH population Linfen 5064 × Nongda 3338.

	**TSS**	**FSS**	**TSSS**	**BSSS**
FSS	0.815^**^			
TSSS	0.271^**^	−0.207^**^		
BSSS	0.328^**^	−0.073	0.067	
GNS	0.336^**^	0.630^**^	−0.437^**^	−0.162^*^

Significance level: ** and * indicate p < 0.01 and 0.05, respectively.

### Linkage Map Construction

In total, 841 SNP markers were used for constructing the LN genetic map. The map had 21 linkage groups, a total length of 3045.86 cM, and an average interval distance of 3.62 cM. The D genome had the lowest marker coverage, especially for chromosomes 5D and 6D. The maps of the A, B, and D genomes had, respectively, lengths of 1324.20, 1322.53, and 399.14 cM and densities of 3.99, 3.28, and 3.77 cM/marker (Supplementary Table S2).

### QTL for Spikelet Number per Spike

A total of 64 QTL for TSS, FSS, TSSS, and BSSS were detected on 18 chromosomes (Supplementary Table S3) with 13 stable QTL identified (Table 3). QTL were found on all chromosomes except 1D, 6D, and 7D (Supplementary Table S3). The QTL explained 3.91–19.51% of the phenotypic variation in different environments. Linfen 5064 alleles contributed 30 of the 64 QTL, and Nongda 3338 contributed 34 alleles. Nine stable QTL, *Qtss.saw-2B.1, Qtss.saw-2B.2, Qtss.saw-3B, Qtss.saw-4A.1, Qtss.saw-5A.1, Qtss.saw-5D, Qfss.saw-2B.2, Qbsss.saw-2B.2*, and *Qbsss.saw-5A.1* were detected in more than three environments and with BLUP values. Except for *Qtss.saw-3B, Qtss.saw-5A.1*, and *Qbsss.saw-5A.1*, the other six QTL explained more than 10% of the phenotypic variance and thus can be considered major stable QTL. The additive effect showed that the alleles of *Qtss.saw-5A.1* and *Qtss.saw-5D* that increased TSS in grain were from Nongda 3338. The six stable QTL *Qtss.saw-2B.1, Qtss.saw-2B.2, Qtss.saw-3B, Qtss.saw-4A.1, Qfss.saw-2B.2*, and *Qbsss.saw-2B.2* carried positive alleles from Linfen 5064. *Qtss.saw-2B.2, Qfss.saw-2B.2*, and *Qbsss.saw-2B.2* were co-located in the *2B_54768734*-*2B_76515060* interval.

**Table 3:** Stable quantitative trait loci (QTL) detected for total spikelet number per spike (TSS), base sterile spikelet number per spike (BSSS), fertile spikelet number per spike (FSS), and grain number per spike (GNS) in the Linfen 5064 × Nongda 3338-derived doubled haploid population.

**Traits**	**QTL**	**Trial**	**Chr**.	**Peak marker**	**Left marker**	**Right marker**	**Genetic distance (cM)**	**LOD**	***R*** **^2^ (%)**	**Add**
TSS	*Qtss.saw-2B.1*	20YC	2B	*2B_712761198*	*2B_712761198*	*2B_690211134*	188.803-191.932	5.75	11.75	0.41
		20HC	2B	*2B_690211134*	*2B_712761198*	*2B_690211134*	188.803–191.932	4.49	8.69	0.39
		20YD	2B	*2B_690211134*	*2B_712761198*	*2B_690211134*	188.803–191.932	3.00	5.32	0.26
		21YD	2B	*2B_690211134*	*2B_712761198*	*2B_690211134*	188.803–191.932	2.76	4.59	0.27
	*Qtss.saw−2B.2*	19YD	2B	*2B_76515060*	*2B_76515060*	*2B_54768734*	278.205–296.593	3.60	5.96	0.27
		20YD	2B	*2B_58866091*	*2B_76515060*	*2B_54768734*	278.205–296.593	6.97	12.99	0.40
		21YD	2B	*2B_58866091*	*2B_76515060*	*2B_54768734*	278.205–296.593	6.60	12.52	0.43
		19YC	2B	*2B_58866091*	*2B_76515060*	*2B_54768734*	278.205–296.593	7.53	13.89	0.51
		20YC	2B	*2B_54768734*	*2B_76515060*	*2B_54768734*	278.205–296.593	4.99	10.66	0.39
		21YC	2B	*2B_58866091*	*2B_76515060*	*2B_54768734*	278.205–296.593	7.33	12.02	0.41
		19HC	2B	*2B_54768734*	*2B_76515060*	*2B_54768734*	278.205–296.593	5.76	13.17	0.59
		20HC	2B	*2B_54768734*	*2B_76515060*	*2B_54768734*	278.205–296.593	2.64	5.81	0.32
		21HC	2B	*2B_53026013*	*2B_76515060*	*2B_54768734*	278.205–296.593	5.20	9.55	0.37
		BLUP	2B	*2B_54768734*	*2B_76515060*	*2B_54768734*	278.205–296.593	8.73	18.45	0.37
	*Qtss.saw-3B*	19YC	3B	*3B_586733548*	*3B_586733548*	*3B_592271369*	39.632–42.79	3.46	6.07	0.34
		21YC	3B	*3B_586733548*	*3B_586733548*	*3B_592271369*	39.632–42.79	2.72	4.74	0.25
		BLUP	3B	*3B_586733548*	*3B_586733548*	*3B_592271369*	39.632–42.79	3.41	5.42	0.20
	*Qtss.saw-4A.1*	19HC	4A	*4A_119796282*	*4A_466206488*	*4A_200909913*	42.02–43.583	5.51	9.52	0.50
		21HC	4A	*4A_200909913*	*4A_444151741*	*4A_290138679*	42.541–44.625	3.95	7.27	0.51
		21YD	4A	*4A_444151741*	*4A_466206488*	*4A_200909913*	42.02–43.583	6.50	11.90	0.33
		BLUP	4A	*4A_444151741*	*4A_466206488*	*4A_200909913*	42.02–43.583	4.26	6.34	0.23
	*Qtss.saw−5A.1*	21HC	5A	*5A_455140212*	*5A_456278473*	*5A_455140212*	182.321–184.952	3.03	5.42	−0.27
		20YC	5A	*5A_455140212*	*5A_456278473*	*5A_455140212*	182.321–184.952	2.93	5.47	−0.28
		21YC	5A	*5A_455140212*	*5A_456278473*	*5A_455140212*	182.321–184.952	3.41	6.71	−0.29
		BLUP	5A	*5A_455140212*	*5A_456278473*	*5A_455140212*	182.321–184.952	2.58	3.91	−0.17
	*Qtss.saw-5D*	19YD	5D	*5D_147564473*	*5D_314429199*	*5D_147564473*	5.748–12.53	7.10	12.77	−0.39
		20YD	5D	*5D_147564473*	*5D_314429199*	*5D_147564473*	5.748–12.53	4.10	7.12	−0.29
		BLUP	5D	*5D_147564473*	*5D_314429199*	*5D_147564473*	5.748–12.53	4.82	7.96	−0.24
FSS	*Qfss.saw-2B.2*	19YD	2B	*2B_76515060*	*2B_76515060*	*2B_54768734*	278.205–296.593	3.08	8.26	0.46
		20YD	2B	*2B_76515060*	*2B_76515060*	*2B_54768734*	278.205–296.593	6.60	15.65	0.47
		19YC	2B	*2B_76515060*	*2B_76515060*	*2B_54768734*	278.205–296.593	7.06	13.69	0.50
		21YC	2B	*2B_76515060*	*2B_76515060*	*2B_54768734*	278.205–296.593	3.47	6.29	0.36
		19HC	2B	*2B_76515060*	*2B_76515060*	*2B_54768734*	278.205–296.593	3.81	8.44	0.47
		BLUP	2B	*2B_76515060*	*2B_76515060*	*2B_54768734*	278.205–296.593	6.93	17.70	0.39
BSSS	*Qbsss.saw−2B.2*	19YD	2B	*2B_76515060*	*2B_76515060*	*2B_54768734*	278.205–296.593	11.02	19.51	0.37
		20YC	2B	*2B_76515060*	*2B_76515060*	*2B_54768734*	278.205–296.593	3.30	6.80	0.18
		19HC	2B	*2B_60980426*	*2B_76515060*	*2B_54768734*	278.205–296.593	3.49	6.84	0.13
		20HC	2B	*2B_60980426*	*2B_76515060*	*2B_54768734*	278.205–296.593	2.87	4.86	0.11
		BLUP	2B	*2B_76515060*	*2B_76515060*	*2B_54768734*	278.205–296.593	6.96	11.12	0.11
	*Qbsss.saw−5A.1*	19HC	5A	*5A_682703894*	*5A_682703894*	*5A_684699297*	0–6.813	3.15	6.46	−0.12
		20HC	5A	*5A_682703894*	*5A_682703894*	*5A_684699297*	0–6.813	3.98	7.37	−0.14
		19YC	5A	*5A_684699297*	*5A_682703894*	*5A_684699297*	0–6.813	3.79	8.64	−0.17
		BLUP	5A	*5A_682703894*	*5A_682703894*	*5A_684699297*	0–6.813	5.35	8.68	−0.10
GNS	*Qgns.saw−5B.2*	19YD	5B	*5B_603868252*	*5B_610798888*	*5B_654359522*	275.112–310.501	3.73	5.34	−2.06
		21YC	5B	*5B_654359522*	*5B_610798888*	*5B_654359522*	275.112–310.501	3.30	7.20	−2.41
		BLUP	5B	*5B_603868252*	*5B_610798888*	*5B_654359522*	275.112–310.501	4.31	8.21	−1.17
	*Qgns.saw-7A.1*	19YD	7A	*7A_658134960*	*7A_657918003*	*7A_658134960*	112.675–113.208	2.78	4.55	1.79
		19YC	7A	*7A_657918003*	*7A_657918003*	*7A_675589691*	112.675–113.208	2.83	5.42	1.86
		20YC	7A	*7A_657918003*	*7A_657918003*	*7A_675589691*	112.675–136.68	3.25	6.80	1.99
		21HC	7A	*7A_657918003*	*7A_657918003*	*7A_675589691*	112.675–136.68	5.19	9.94	2.13
		BLUP	7A	*7A_658134960*	*7A_657918003*	*7A_658134960*	112.675–113.208	4.06	9.44	1.20
	*Qgns.saw-4D*	21YC	4D	*4D_15772687*	*4D_15772687*	*4D_48697668*	0–7.629	4.27	7.66	−2.46
		20YC	4D	*4D_48697668*	*4D_15772687*	*4D_48697668*	0–7.629	3.43	6.71	−2.02
		19HC	4D	*4D_193777167*	*4D_15772687*	*4D_48697668*	0–7.629	5.63	10.80	−2.44
	*Qgns.saw-1A*	21YD	1A	*1A_567714120*	*1A_567714120*	*1A_568327780*	0–1.46	3.72	6.60	−2.03
		21HC	1A	1A_568327780	1A_567714120	1A_568327780	0–1.46	5.79	11.16	−0.92
		21YC	1A	1A_568327780	1A_567714120	1A_568327780	0–1.46	2.56	4.47	−2.25
		BLUP	1A	1A_567714120	1A_567714120	1A_568327780	0–1.46	2.75	5.25	−1.88

### QTL for Grain Number per Spike

For GNS, 16 QTL were detected and these QTL explained 4.18–15.83% of the phenotypic variance (Supplementary Table S3). Four stable QTL, *Qgns.saw-5B.2, Qgns.saw-7A.1, Qgns.saw-4D*, and *Qgns.saw-1A*, explaining 4.47–11.16% of the phenotypic variance were identified in more than three environments and with BLUP values (Table 3). The additive effect of *Qgns.saw-7A.1* was from Linfen 5064 indicating that Linfen 5064 contributed the allele for increased GNS. No stable QTL clusters for GNS and spikelet number per spike were detected on the same chromosome, indicating that the QTL of GNS were most likely independent of spikelet number per spike and therefore have great potential in wheat breeding.

### QTL Validation

**Figure 1:**
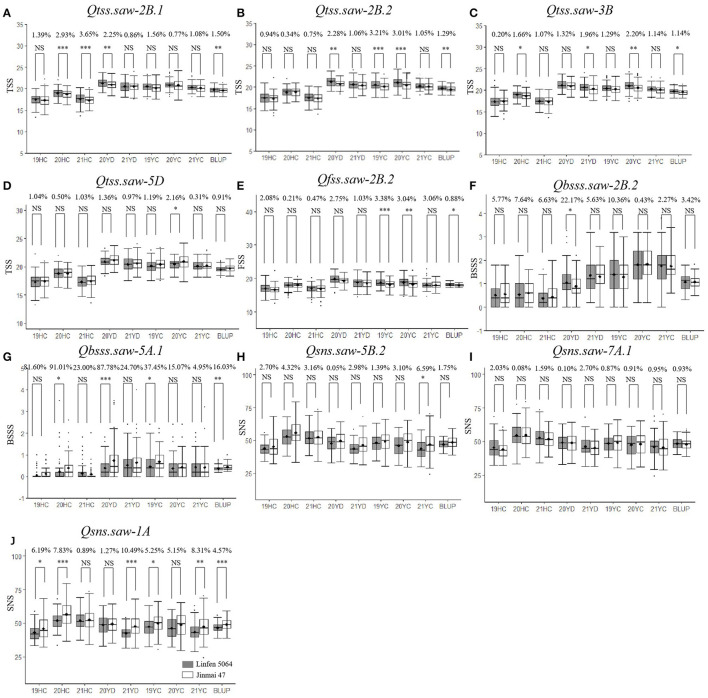
Validation of 10 stable quantitative trait loci (QTL) in LJ population. Effects of **(A)** Qtss.saw-2B.1, **(B)** Qtss.saw-2B.2, **(C)** Qtss.saw-3B, **(D)** Qtss.saw-5D, **(E)** on total spikelet number per spike (TSS) and effects of Qfss.saw-2B.2, **(F)** on fertile spikelet number per spike (FSS) and effects of Qbsss.saw-2B.2, **(G)** Qbsss.saw-5A.1, **(H)** on base sterile spikelet number per spike (BSSS) and effects of Qsns.saw-5B.2, **(I)** Qsns.saw-7A.1, **(J)** and Qsns.saw-1A on grain number per spike (GNS). ^*^, ^**^, ^***^ and NS represent *P* < 0.05, *P* < 0.01, *P* < 0.001 and no significant difference,respectively.

To further validate the stable QTL, the peak SNPs for each were used to evaluate their effects on corresponding traits in the LJ population. The peak markers for *Qtss.saw-4A.1, Qtss.saw-5A.1*, and *Qgns.saw-4D* were not polymorphic between the LJ parents, and thus could not be evaluated. The remaining 10 QTL were evaluated. The effect of *Qtss.saw-5D, Qgns.saw-5B.2, Qgns.saw-7A.1*, and *Qbsss.saw-2B.2* did not differ significantly between the two groups in the LJ population (Figure 1). The effect of other six QTL, *Qtss.saw-2B.1, Qtss.saw-2B.2, Qfss.saw-2B.2, Qtss.saw-3B, Qbsss.saw-5A.1*, and *Qgns.saw-1A*, were highly significant (*P* < 0.05) in more than three environments. According to marker profiles of *Qtss.saw-2B.1, Qtss.saw-2B.2*, and *Qtss.saw-3B*, lines with homozygous alleles from Linfen 5064 had significantly higher (*P* < 0.05) values for TSS than those from Nongda 3338 and the difference ranged from 1.14 to 3.65%. The *Qtss.saw-2B.2* lines homozygous for the Linfen 5064 alleles had significantly higher phenotypic values than those with the Jinmai 47 alleles irrespective of QTL region, with differences in TSS ranging from 1.29 to 3.21%. Lines with the positive allele from *Qfss.saw-2B.2* had significantly greater FSS ranging from 0.88 to 3.38%, corresponding to 0.15–0.62 more spikelets than the lines with the alternate allele.

### Analyses of Additive Effects of the Major QTL

**Figure 2:**
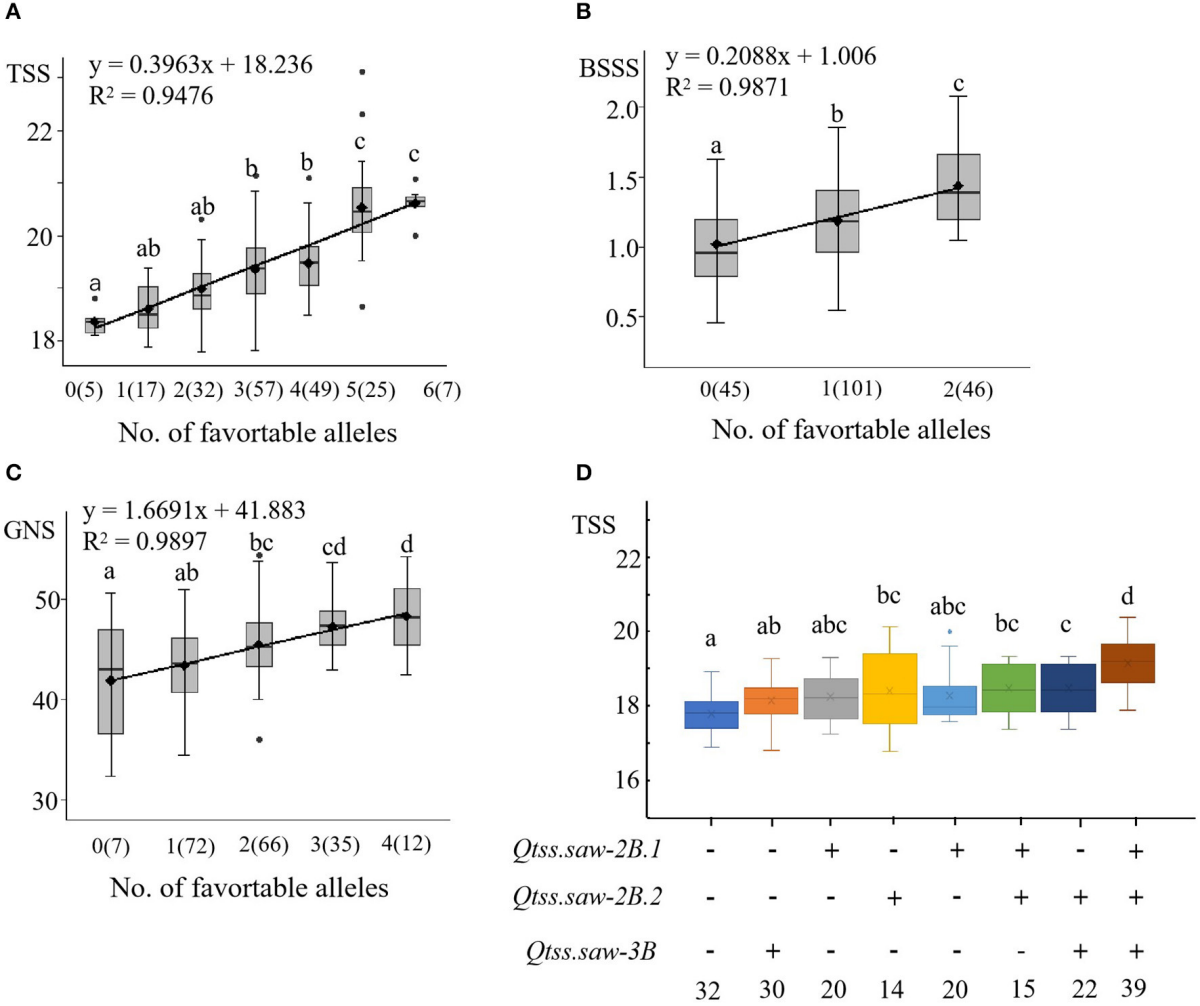
Linear regressions between the number of TSS, BSSS, and GNS **(A–C)** and additive effects of the QTL for TSS **(D)** in the LN population. The numbers of lines carrying the corresponding number of favorable alleles are shown in brackets. The letter above the bars indicated comparisons result at the significant level 0.05, respectively. Plus and minus represent lines with and without the positive alleles of the target QTL based on the flanking markers and the corresponding QTL.

In the LN population, we detected six stable QTL for TSS (*Qtss.saw-2B.1, Qtss.saw-2B.2, Qtss.saw-3B, Qtss.saw-4A.1, Qtss.saw-5A.1*, and *Qtss.saw-5D*), *two* stable QTL for BSSS (*Qbsss.saw-2B.2* and *Qbsss.saw-5A.1*), and four stable QTL for GNS (*Qgns.saw-5B.2, Qgns.saw-7A.1, Qgns.saw-4D*, and *Qgns.saw-1A*) (Table 3). The additive effects of these QTL on corresponding traits were analyzed based on linked markers. The average corresponding trait values increased as the number of positive alleles increased (Figures 2A–C). Lines with favorable alleles at all the six QTL regions had an average TSS increase of 2.25 vs. those possessing contrasting alleles (Supplementary Table S4, Figure 2A). Lines with both the positive alleles had significantly increased values for BSSS (Figure 2B). The combination of positive alleles from *Qgns.saw-5B.2, Qgns.saw-7A.1, Qgns.saw-4D*, and *Qgns.saw-1A* had the largest effect on GNS (Supplementary Table S4, Figure 2C).

*Qtss.saw-2B.1, Qtss.saw-2B.2*, and *Qtss.saw-3B* were validated in the LJ population, and the positive alleles of three QTL were derived from Linfen 5064, the additive effects on each corresponding trait were analyzed based on linked markers (Supplementary Table S5, Figure 2D). The combination of positive alleles from *Qtss.saw-2B.1, Qtss.saw-2B.2*, and *Qtss.saw-3B* (7.33%, 1.38) had the largest effect on TSS. Compared with lines lacking positive alleles for increased TSS, the positive allele from *Qtss.saw-2B.2* significantly increased TSS by 3.30%, which was higher than that for the other single positive alleles of *Qtss.saw-2B.1* (1.92%, 0.36) and *Qtss.saw-3B* (2.45%, 0.46). DH lines with both *Qtss.saw-2B.1* and *Qtss.saw-3B* positive alleles significantly increased TSS (2.56%, 0.48) less than that of DH lines with single positive alleles of *Qtss.saw-2B.2* (3.30%, 0.62). These results indicated that the positive allele of *Qtss.saw-2B.2* from Linfen 5064 has a larger effect on TSS.

### Distribution of Linfen 5064 Favorable Alleles Across Cultivars

**Figure 3:**
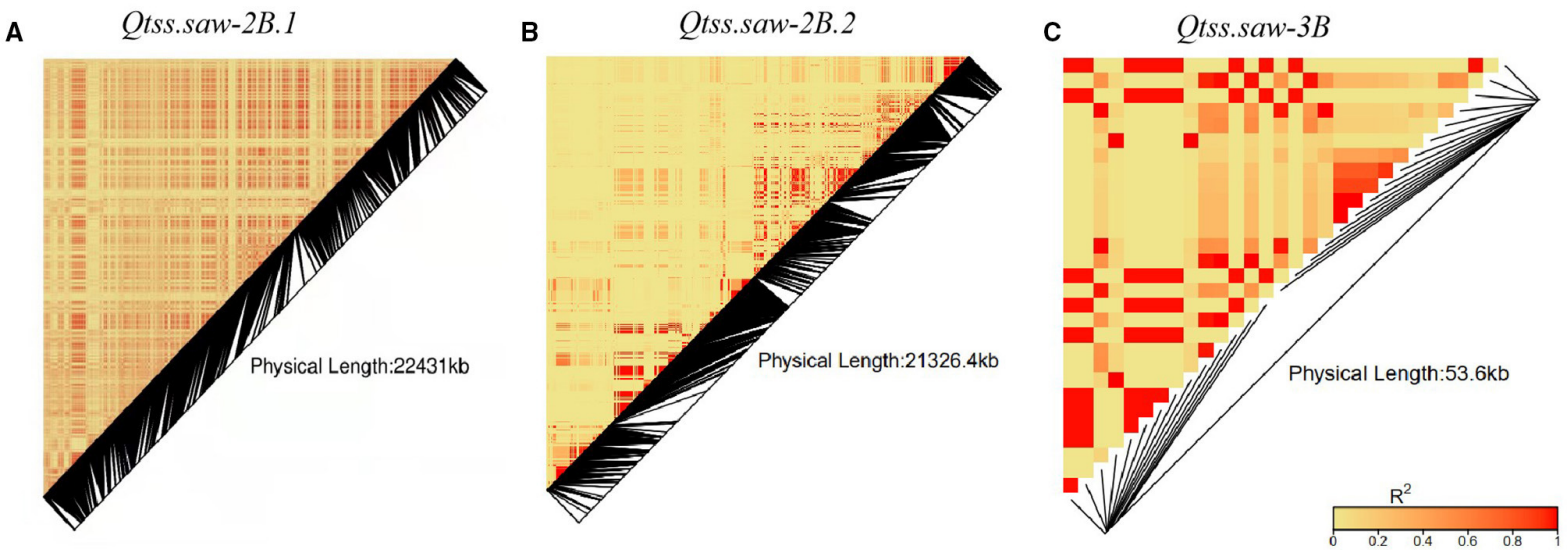
Linkage disequilibrium heatmap of three target QTL regions **(A)** Qtss.saw-2B.1, **(B)** Qtss.saw-2B.2, and **(C)** Qtss.saw-3B.

The three stable QTL *Qtss.saw-2B.1, Qtss.saw-2B.2*, and *Qtss.saw-3B* were detected in more than three environments and were validated in the LJ population. The additive effects of these QTL were from Linfen 5064. Based on the resequencing of 145 wheat cultivars, linkage disequilibrium analysis was performed to assess variation sites within three target QTL regions (Figure 3). *Qtss.saw-2B.1, Qtss.saw-2B.2*, and *Qtss.saw-3B* had high recombination rates corresponding to recombination hotspot areas. Therefore, for three QTL the distribution of favorable alleles from Linfen 5064 was analyzed in 145 landmark cultivars (Table 4). The favorable alleles of Linfen 5064 for *Qtss.saw-2B.2* had a lower proportion in the Chinese landraces (CL) (44%) and introduced modern cultivars (IMC) (45%), but a higher proportion in the modern Chinese cultivars (MCC) (77%). Therefore, the favorable alleles of Linfen 5064 at the *Qtss.saw-2B.2* locus were selected because of their value in breeding new Chinese cultivars. *Qtss.saw-2B.1* and *Qtss.saw-3B* with the positive Linfen 5064 alleles were less frequent in Chinese landmark cultivars (29.66 and 15.86%, respectively), indicating that *Qtss.saw-3B* landmark alleles tended to be replaced during breeding by the Linfen 5064 alleles.

**Table 4:** The proportion of the Linfen 5064 favorable alleles detected in 145 cultivars *Qtss.saw-2B.1, Qtss.saw-2B.2*, and *Qtss.saw-3B*.

**QTL**	**Parent**	**Allele**	**CL (%)**	**IMC (%)**	**MC (%)**	**Cultivars (%)**
*Qtss.saw-2B.1*	Linfen 5064	A	20.00	20.00	34.00	29.66
		G	76.00	35.00	43.00	47.59
*Qtss.saw-2B.2*	Linfen 5064	A	44.00	45.00	77.00	66.90
		C	36.00	50.00	18.00	25.52
*Qtss.saw-3B*	Linfen 5064	C	8.00	10.00	19.00	15.86
		T	92.00	90.00	81.00	84.14

*CL, Chinese landraces, IMC, introduced modern cultivars; MCC, modern Chinese cultivars*.

### Genes Identified in the Major QTL

A series of orthologous GNS-related genes have been cloned in rice (Huang et al., 2009; Kyoko et al., 2009; Qiao et al., 2011; Gao et al., 2016) and wheat (Jiang et al., 2015; Zhang et al., 2015; Shao et al., 2017; Muqaddasi et al., 2019; Rehman et al., 2019), these genes always showed conserved functions across grass species (Valluru et al., 2014). Based on the result of local-blast browse through the IWGSC reference sequence, no homologs of the above genes were found in the physical regions of 690.21–712.76 Mb on 2BL and 586.73–592.27 Mb on 3BL in wheat. It indicated that there might be novel genes related to GNS among the two QTL, thus, these QTL were chosen for further analysis. *Qtss.saw-2B.1* was in the interval 690.21–712.76 Mb on 2BL and where 260 genes have been found in the variety Chinese Spring (CS) (Supplementary Table S6). Gene annotation, expression pattern, and orthologous gene analysis indicate that three genes are likely involved in spike development (Supplementary Table S6, Supplementary Figure S1). The function of *TraesCS2B02G500100, TraesCS2B02G500200*, and *TraesCS2B02G500300* are annotated as a series of molecular signals generated by the binding of the plant hormone abscisic acid to a receptor and ending with modulation of a cellular process. *Qtss.saw-3B* has 20 genes in CS and 13 common predicated genes between CS and rice (Supplementary Table S7). The genes were not preferentially expressed in spike and grain (Supplementary Figure S2).

## Discussion

### Linfen 5064 Possess Favorable Key Genomic Regions

Analyzing founder parents at the whole genome level and studying the genome regions of the founder parents of high value is important for wheat breeding, especially molecular marker-assisted breeding. As a founder parent, Linfen 5064 has greatly contributed to wheat breeding in China. The high-quality characteristics of Linfen 5064 are derived from the spring wheat SARICF74 introduced from the Centro Internacional de Mejoramiento de Maizy Trigo (CIMMYT). Linfen 5064 was selected from a cross of SARICF74 and Linfen 5694 for early maturity and good agronomic traits. In this study, two DH populations were constructed with Linfen 5064 as the female parent and with Nongda 3338 and Jinmai 47 as male parents. A total of 13 stable QTL were identified through the investigation of spike traits in three field locations over 3 years. Seven stable QTL carried positive alleles from Linfen 5064. For spikelet number per spike, the additive effect of *Qtss.saw-2B.1, Qtss.saw-2B.2, Qtss.saw-3B, Qtss.saw-4A.1, Qfss.saw-2B.2*, and *Qbsss.saw-2B.2* were from Linfen 5064. And except for *Qtss.saw-4A.1 and Qbsss.saw-2B.2*, other QTL were validated in the LJ population. The QTL *Qtss.saw-2B.2, Qfss.saw-2B.2*, and *Qbsss.saw-2B.2* were located in the same region. Therefore, *Qtss.saw-2B.1, Qtss.saw-2B.2*, and *Qtss.saw-3B* were the most important regions of Linfen 5064 controlling spikelet number per spike. Lines with the positive allele from *Qtss.saw-2B.2* significantly increased TSS by 3.30%, which is higher than other single positive alleles of either *Qtss.saw-2B.1* (1.92%) or *Qtss.saw-3B* (2.45%). The region *Qtss.saw-2B.2* from Linfen 5064 had the larger effect on TSS and was present in 66.89% of Chinese landmark cultivars tested. For GNS, the only positive effect from a Linfen 5064 allele was from *Qgns.saw-7A.1*. The positive effects of *Qgns.saw-5B.2, Qgns.saw-4D*, and *Qgns.saw-1A* alleles were from Nongda 3338. The effect of *Qgns.saw-1A* was validated in the LJ population. These results indicate that this allele was unfavorable, but through breeding, improvement was made for the trait in Linfen 5064 presumably from contributions from other loci. This study examined the QTL of Linfen 5064 for GNS and analyzed the characteristics of genetic effects of related regions. These results further clarify the genetic contribution and intrinsic value of Linfen 5064 to GNS and provide a reference for future founder parent utilization and molecular breeding.

### *Qtss.saw-2B.1* and *Qtss.saw-3B* Are Novel Loci for Wheat Spike-Related Traits

To compare the intervals of the 13 QTL detected with those identified previously, we physically mapped these QTL on target chromosomes in CS. The QTL *Qtss.saw-5D* for TSS is physically located between 147.56 and 314.43 Mb on 5D (Table 3). It overlapped with a major QTL *QSN.caas-5DL* found in wheat by Li et al. (2018). *Qgns.saw-7A.1* was located between 657.92 and 675.59 Mb on chromosome 7AL (Table 3). This region has QTL-rich clusters for wheat yield component traits. *QSn-7A.2* (Fan et al., 2019), *Qmt.tamu.7A.1* (Assanga et al., 2017), *QTgw.cau-7A.4* (Guan et al., 2018), and *Qkns.caas-7AL* (Li et al., 2018) overlap with *Qgns.saw-7A.1*. Likewise, *TaAPO-A1* is in this cluster, namely, *Qkns.caas-7AL, QGne.nfcri-7A*, and *QGns.cau-7A.5* for Kernel number per spike, so it probably is the candidate gene of these QTL (Cao et al., 2020). *TaAPO-A1* is orthologous to *APO1*, a rice gene that positively controls spikelet number on panicles (Muqaddasi et al., 2019). *Qgns.saw-4D* was located within 15.77–48.70 Mb on chromosome 4DS (Table 3). Comparative analysis revealed that this locus overlaps *TB-D1* (Dixon et al., 2018), *Rht-D1* (Peng et al., 1999), *QTKW-4D-AN* (Mohler et al., 2016), *QGn.nau-4D* (Jia et al., 2013), *QTgw-4D*, and *QGns-4D* (Liu et al., 2014), suggesting this region is a QTL-rich cluster for wheat yield component traits. *Qtss.saw-2B.2, Qfss.saw-2B.2*, and *Qbsss.saw-2B.2* were co-located in the interval of *2B_54768734*-*2B_76515060* and physically mapped to 54.77–76.52 Mb on 2BS. This region has the *Ppd-B1* gene which is a key component in the photoperiod regulatory flowering pathway (Beales et al., 2007; Nishida et al., 2013) and is associated with flag leaf size and grain yield (Kirby, 1992; Snape et al., 2001; Foulkes et al., 2004). No stable QTL have been reported previously overlapped with the other stable QTL from this study, *Qtss.saw-2B.1, Qtss.saw-3B, Qtss.saw-5A*, and *Qgns.saw-5B.2*. Both *Qtss.saw-2B.1* and *Qtss.saw-3B* had significant effects on TSS and GNS that were detected in the validation population. Therefore, *Qtss.saw-2B.1* and *Qtss.saw-3B* are likely novel loci for TSS. Therefore, spikelet development of wheat is a complex process, which is regulated by different types of genes. With the development of biotechnology, combining multiple technologies to analyze the development of GNS will help clarify the formation mechanism of GNS.

### New Genes Were Identified in the Interval of the Stable QTL to Control Spike-Related Traits

Genes related to spike traits can be divided into two categories. The first category is flowering time (FT) genes which have significant effects on grain yield, namely, *Vrn1, Vrn2*/*ZCCT1, Vrn3*, and *Ppd-D1* (Cuthbert et al., 2008; Zhou et al., 2017; Guan et al., 2018). Other genes were mainly involved in spike differentiation which influenced the number of grains per spike by regulating the rate and direction of differentiation. For example, *aberrant panicle organization 1 (APO1)* controls cell proliferation of the rice meristem, leading to the reduction of the primary and secondary branches of the panicle, thereby affecting panicle development (Kyoko et al., 2009). In addition, some genes can control panicle morphogenesis by regulating hormone and protein expression during rice growth (Huang et al., 2009; Qiao et al., 2011; Gao et al., 2016). *BG1* regulates auxin transport and increases biomass, grain number per spike, and grain size to increase yield (Liu et al., 2015). In this study, we find three new genes for controlling spike-related traits. *TraesCS2B02G500100, TraesCS2B02G500200*, and *TraesCS2B02G500300* and involved the phytohormone regulatory and ubiquitin proteasoma. In the next step, we will fine-mapping these QTL which will help explain the formation and development of GNS in wheat and develop linked molecular markers for use by breeders.

## Data Availability Statement

The datasets presented in this study can be found in online repositories. The names of the repository/repositories and accession number(s) can be found in the article/Supplementary Material.

## Author Contributions

JZhe, WD, JuW, and LQ designed the experiment and developed the original manuscript. LQ, HL, JZha, XZ, JiW, and BW performed the field experiments. LQ, HL, XZ, WD, and JZhe performed the phenotypic data analysis and the QTL detection. WD, JuW, and JZhe revised the manuscript. All authors approved the submitted version of the manuscript.

## Funding

This study was supported by the State Key Laboratory of Integrative Sustainable Dryland Agriculture (in preparation), the Shanxi Agricultural University (No. 202105D121008-2-1), the Shanxi Scholarship Council of China (2020-159), the Shanxi Province Research Program (20210302124505), and the Agricultural Science Research of Shanxi Academy of Agricultural Sciences (YCX2020YQ47, YCX2020YQ34, and YZGC013).

## Conflict of Interest

The authors declare that the research was conducted in the absence of any commercial or financial relationships that could be construed as a potential conflict of interest.

## Publisher's Note

All claims expressed in this article are solely those of the authors and do not necessarily represent those of their affiliated organizations, or those of the publisher, the editors and the reviewers. Any product that may be evaluated in this article, or claim that may be made by its manufacturer, is not guaranteed or endorsed by the publisher.
