# Supplementary figures and images for: Analysis of Genetic Regions Related to Field Grain Number per Spike From Chinese Wheat Founder Parent Linfen 5064
Source: Front Plant Sci. 2022 Jan 5;12:808136. doi: 10.3389/fpls.2021.808136 (PMC8769526; doi:10.3389/fpls.2021.808136)

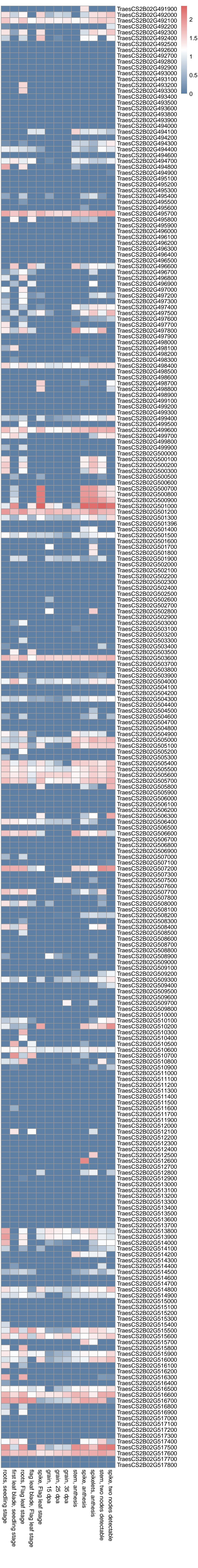

Supplement: Supplementary file 1 [file Data_Sheet_1.PDF]

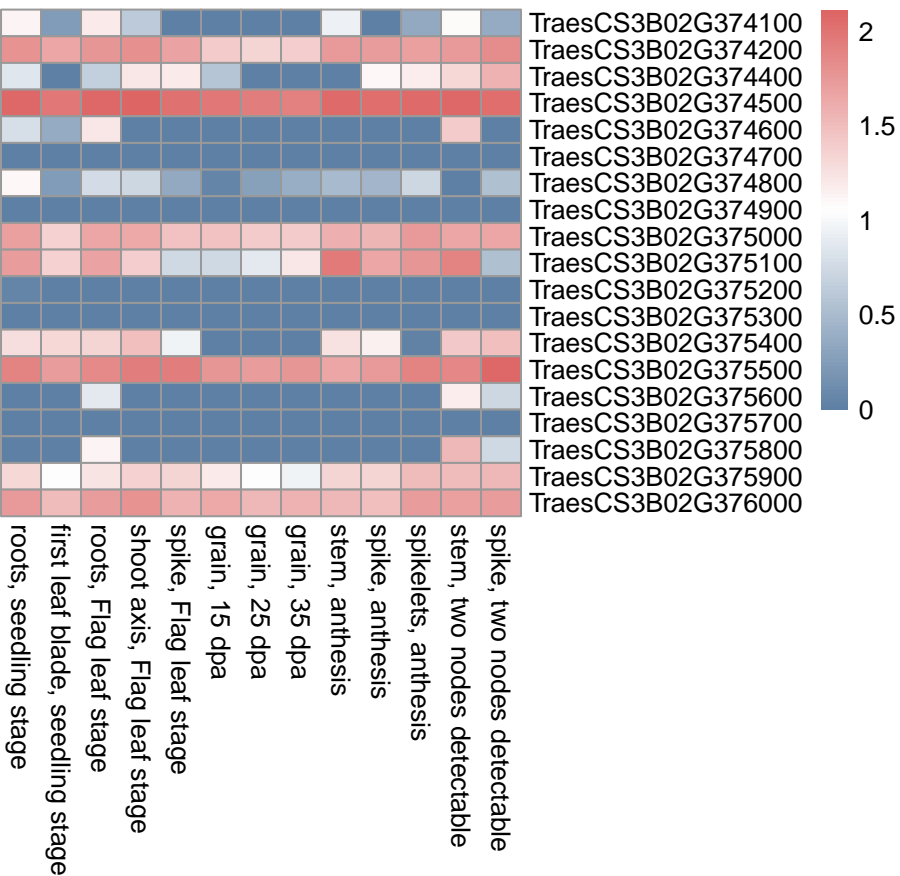

Supplement: Supplementary file 2 [file Data_Sheet_2.PDF]
