# Supplementary material for: Analysis of Genetic Regions Related to Field Grain Number per Spike From Chinese Wheat Founder Parent Linfen 5064
Source: Front Plant Sci. 2022 Jan 5;12:808136. doi: 10.3389/fpls.2021.808136 (PMC8769526; doi:10.3389/fpls.2021.808136)
Supplement: Supplementary file 3 [file Table_1.docx]

**Supplementary Tables**

**Table S1.** Correlation coefficients for five spike related traits among nine trials

| Traits | Trial | 19HC | 20HC | 21HC | 19YD | 20YD | 21YD | 19YC | 20YC |
| --- | --- | --- | --- | --- | --- | --- | --- | --- | --- |
| TSS | 20HC | 0.624** |  |  |  |  |  |  |  |
|  | 21HC | 0.525** | 0.504** |  |  |  |  |  |  |
|  | 19YD | 0.606** | 0.579** | 0.456** |  |  |  |  |  |
|  | 20YD | 0.528** | 0.468** | 0.442** | 0.606** |  |  |  |  |
|  | 21YD | 0.564** | 0.525** | 0.341** | 0.630** | 0.557** |  |  |  |
|  | 19YC | 0.490** | 0.408** | 0.434** | 0.556** | 0.648** | 0.586** |  |  |
|  | 20YC | 0.527** | 0.547** | 0.401** | 0.657** | 0.606** | 0.527** | 0.642** |  |
|  | 21YC | 0.537** | 0.560** | 0.406** | 0.654** | 0.598** | 0.578** | 0.597** | 0.663** |
| FSS | 20HC | 0.560** |  |  |  |  |  |  |  |
|  | 21HC | 0.454** | 0.367** |  |  |  |  |  |  |
|  | 19YD | 0.262** | 0.410** | 0.213** |  |  |  |  |  |
|  | 20YD | 0.349** | 0.333** | 0.283** | 0.217** |  |  |  |  |
|  | 21YD | 0.467** | 0.479** | 0.287** | 0.344** | 0.464** |  |  |  |
|  | 19YC | 0.456** | 0.340** | 0.296** | 0.230** | 0.468** | 0.383** |  |  |
|  | 20YC | 0.355** | 0.339** | 0.263** | 0.359** | 0.278** | 0.371** | 0.468** |  |
|  | 21YC | 0.473** | 0.423** | 0.173* | 0.438** | 0.310** | 0.369** | 0.427** | 0.385** |
| TSSS | 20HC | 0.481** |  |  |  |  |  |  |  |
|  | 21HC | 0.181* | 0.281** |  |  |  |  |  |  |
|  | 19YD | 0.394** | 0.405** | 0.134 |  |  |  |  |  |
|  | 20YD | 0.225** | 0.364** | -0.072 | 0.285** |  |  |  |  |
|  | 21YD | 0.216** | 0.248** | 0.258** | 0.246** | 0.200** |  |  |  |
|  | 19YC | 0.240** | 0.391** | 0.264** | 0.406** | 0.163* | 0.183* |  |  |
|  | 20YC | 0.332** | 0.320** | 0.214** | 0.435** | 0.102 | 0.185* | 0.498** |  |
|  | 21YC | 0.403** | 0.525** | 0.177* | 0.491** | 0.282** | 0.304** | 0.519** | 0.494** |
| BSSS | 20HC | 0.494** |  |  |  |  |  |  |  |
|  | 21HC | 0.331** | 0.303** |  |  |  |  |  |  |
|  | 19YD | 0.195** | 0.239** | 0.243** |  |  |  |  |  |
|  | 20YD | 0.262** | 0.249** | 0.150* | 0.407** |  |  |  |  |
|  | 21YD | 0.341** | 0.258** | 0.093 | 0.219** | 0.243** |  |  |  |
|  | 19YC | 0.372** | 0.328** | 0.250** | 0.238** | 0.330** | 0.375** |  |  |
|  | 20YC | 0.319** | 0.211** | 0.321** | 0.491** | 0.263** | 0.260** | 0.376** |  |
|  | 21YC | 0.339** | 0.320** | 0.295** | 0.242** | 0.303** | 0.211** | 0.317** | 0.298** |
| GNS | 20HC | 0.448** |  |  |  |  |  |  |  |
|  | 21HC | 0.279** | 0.241** |  |  |  |  |  |  |
|  | 19YD | 0.410** | 0.290** | 0.178* |  |  |  |  |  |
|  | 20YD | 0.440** | 0.345** | 0.151* | 0.281** |  |  |  |  |
|  | 21YD | 0.387** | 0.343** | 0.203** | 0.383** | 0.378** |  |  |  |
|  | 19YC | 0.442** | 0.351** | 0.259** | 0.327** | 0.333** | 0.408** |  |  |
|  | 20YC | 0.155* | 0.132 | 0.198** | 0.246** | 0.022 | 0.147* | 0.544** |  |
|  | 21YC | 0.507** | 0.356** | 0.169* | 0.330** | 0.367** | 0.473** | 0.611** | 0.279** |

*Significant at *P* < 0.05; **significant at *P*< 0.01

**Table S2.** Summary of linkage groups derived from linkage analysis with 15K SNP markers in the Linfen 5064×Nongda 3338 derived doubled haploid population.

| Chromosome | Linfen 5064×Nongda 3338 | | |
| --- | --- | --- | --- |
|  | No. of SNP | Length (cM) | Marker Density (cM/marker) |
| 1A | 45 | 111.68 | 2.48 |
| 2A | 35 | 201.09 | 5.75 |
| 3A | 55 | 274.71 | 4.99 |
| 4A | 38 | 160.95 | 4.24 |
| 5A | 58 | 244.15 | 4.21 |
| 6A | 44 | 149.07 | 3.39 |
| 7A | 57 | 182.56 | 3.20 |
| 1B | 43 | 115.95 | 2.70 |
| 2B | 91 | 358.91 | 3.94 |
| 3B | 40 | 119.36 | 2.98 |
| 4B | 32 | 125.04 | 3.91 |
| 5B | 110 | 325.76 | 2.96 |
| 6B | 57 | 136.71 | 2.40 |
| 7B | 30 | 140.79 | 4.69 |
| 1D | 27 | 82.19 | 3.04 |
| 2D | 14 | 101.50 | 7.25 |
| 3D | 16 | 65.26 | 4.08 |
| 4D | 23 | 73.68 | 3.20 |
| 5D | 6 | 13.05 | 2.18 |
| 6D | 3 | 1.16 | 0.39 |
| 7D | 17 | 62.30 | 3.66 |
| A genome | 332 | 1324.20 | 3.99 |
| B genome | 403 | 1322.53 | 3.28 |
| D genome | 106 | 399.14 | 3.77 |
| Total | 841 | 3045.86 | 3.62 |

**Table S3.** Quantitative trait loci (QTL) for five spike related traits detected in all the environments in the DH population.

| Traits | QTL | Trial | Chr. | Peak Marker | Left Marker | Right Marker | Genetic distance (cM) | LOD | *R*2 (%) | Add |
| --- | --- | --- | --- | --- | --- | --- | --- | --- | --- | --- |
| TSS | *Qtss.saw-2B.1* | 20YC | 2B | *2B_712761198* | *2B_712761198* | *2B_690211134* | 188.803-191.932 | 5.75 | 11.75 | 0.41 |
|  |  | 20HC | 2B | *2B_690211134* | *2B_712761198* | *2B_690211134* | 188.803-191.932 | 4.49 | 8.69 | 0.39 |
|  |  | 20YD | 2B | *2B_690211134* | *2B_712761198* | *2B_690211134* | 188.803-191.932 | 3.00 | 5.32 | 0.26 |
|  |  | 21YD | 2B | *2B_690211134* | *2B_712761198* | *2B_690211134* | 188.803-191.932 | 2.76 | 4.59 | 0.27 |
|  | *Qtss.saw-2B.2* | 19YD | 2B | *2B_76515060* | *2B_76515060* | *2B_54768734* | 278.205-296.593 | 3.60 | 5.96 | 0.27 |
|  |  | 20YD | 2B | *2B_58866091* | *2B_76515060* | *2B_54768734* | 278.205-296.593 | 6.97 | 12.99 | 0.40 |
|  |  | 21YD | 2B | *2B_58866091* | *2B_76515060* | *2B_54768734* | 278.205-296.593 | 6.60 | 12.52 | 0.43 |
|  |  | 19YC | 2B | *2B_58866091* | *2B_76515060* | *2B_54768734* | 278.205-296.593 | 7.53 | 13.89 | 0.51 |
|  |  | 20YC | 2B | *2B_54768734* | *2B_76515060* | *2B_54768734* | 278.205-296.593 | 4.99 | 10.66 | 0.39 |
|  |  | 21YC | 2B | *2B_58866091* | *2B_76515060* | *2B_54768734* | 278.205-296.593 | 7.33 | 12.02 | 0.41 |
|  |  | 19HC | 2B | *2B_54768734* | *2B_76515060* | *2B_54768734* | 278.205-296.593 | 5.76 | 13.17 | 0.59 |
|  |  | 20HC | 2B | *2B_54768734* | *2B_76515060* | *2B_54768734* | 278.205-296.593 | 2.64 | 5.81 | 0.32 |
|  |  | 21HC | 2B | *2B_53026013* | *2B_76515060* | *2B_54768734* | 278.205-296.593 | 5.20 | 9.55 | 0.37 |
|  |  | BLUP | 2B | *2B_54768734* | *2B_76515060* | *2B_54768734* | 278.205-296.593 | 8.73 | 18.45 | 0.37 |
|  | *Qtss.saw-3B* | 19YC | 3B | *3B_586733548* | *3B_586733548* | *3B_592271369* | 39.632-42.79 | 3.46 | 6.07 | 0.34 |
|  |  | 21YC | 3B | *3B_586733548* | *3B_586733548* | *3B_592271369* | 39.632-42.79 | 2.72 | 4.74 | 0.25 |
|  |  | BLUP | 3B | *3B_586733548* | *3B_586733548* | *3B_592271369* | 39.632-42.79 | 3.41 | 5.42 | 0.20 |
|  | *Qtss.saw-4A.1* | 19HC | 4A | *4A_119796282* | *4A_466206488* | *4A_200909913* | 42.02-43.583 | 5.51 | 9.52 | 0.50 |
|  |  | 21HC | 4A | *4A_200909913* | *4A_444151741* | *4A_290138679* | 42.541-44.625 | 3.95 | 7.27 | 0.51 |
|  |  | 21YD | 4A | *4A_444151741* | *4A_466206488* | *4A_200909913* | 42.02-43.583 | 6.50 | 11.90 | 0.33 |
|  |  | BLUP | 4A | *4A_444151741* | *4A_466206488* | *4A_200909913* | 42.02-43.583 | 4.26 | 6.34 | 0.23 |
|  | *Qtss.saw-5A.1* | 21HC | 5A | *5A_455140212* | *5A_456278473* | *5A_455140212* | 182.321-184.952 | 3.03 | 5.42 | -0.27 |
|  |  | 20YC | 5A | *5A_455140212* | *5A_456278473* | *5A_455140212* | 182.321-184.952 | 2.93 | 5.47 | -0.28 |
|  |  | 21YC | 5A | *5A_455140212* | *5A_456278473* | *5A_455140212* | 182.321-184.952 | 3.41 | 6.71 | -0.29 |
|  |  | BLUP | 5A | *5A_455140212* | *5A_456278473* | *5A_455140212* | 182.321-184.952 | 2.58 | 3.91 | -0.17 |
|  | *Qtss.saw-5D* | 19YD | 5D | *5D_147564473* | *5D_314429199* | *5D_147564473* | 5.748-12.53 | 7.10 | 12.77 | -0.39 |
|  |  | 20YD | 5D | *5D_147564473* | *5D_314429199* | *5D_147564473* | 5.748-12.53 | 4.10 | 7.12 | -0.29 |
|  |  | BLUP | 5D | *5D_147564473* | *5D_314429199* | *5D_147564473* | 5.748-12.53 | 4.82 | 7.96 | -0.24 |
|  | *Qtss.saw-1B* | 20HC | 1B | *1B_343365455* | *1B_474387176* | *1B_314122022* | 105.472-110.193 | 3.90 | 6.15 | -0.33 |
|  | *Qtss.saw-2B.3* | 21YC | 2B | *2B_593335690* | *2B_607399152* | *2B_593335690* | 157.072-158.114 | 7.01 | 13.31 | 0.41 |
|  | *Qtss.saw-2B.4* | BLUP | 2B | *2B_671038545* | *2B_671038545* | *2B_672999249* | 180.428-180.957 | 4.00 | 5.94 | -0.53 |
|  | *Qtss.saw-2D.1* | 19HC | 2D | *2D_521528010* | *2D_502217152* | *2D_521528010* | 41.15-41.671 | 2.70 | 5.16 | -0.36 |
|  | *Qtss.saw-2D.2* | 19YD | 2D | *2D_406969238* | *2D_531701660* | *2D_406969238* | 50.008-62.036 | 3.43 | 6.60 | -0.28 |
|  | *Qtss.saw-2D.3* | 20YC | 2D | *2D_76616365* | *2D_406969238* | *2D_76616365* | 62.036-83.472 | 2.66 | 4.64 | -0.26 |
|  | *Qtss.saw-4A.2* | 20HC | 4A | *4A_591709139* | *4A_552270778* | *4A_597902329* | 63.394-80.229 | 5.24 | 9.26 | 0.41 |
|  | *Qtss.saw-4A.3* | 21YD | 4A | *4A_15121018* | *4A_15121018* | *4A_17717246* | 0-3.357 | 3.05 | 5.37 | -0.33 |
|  | *Qtss.saw-4D.1* | 20HC | 4D | *4D_48697668* | *4D_15772687* | *4D_48697668* | 0-7.629 | 4.16 | 6.56 | 0.34 |
|  | *Qtss.saw-4D.2* | 19YC | 4D | *4D_104889589* | *4D_370155010* | *4D_104889589* | 21.321-23.941 | 2.98 | 5.21 | -0.31 |
|  | *Qtss.saw-5B* | 21YD | 5B | *5B_429637379* | *5B_421696566* | *5B_429637379* | 70.767-75.995 | 4.45 | 7.96 | -0.35 |
|  |  | 19YC | 5B | *5B_429637379* | *5B_421696566* | *5B_429637379* | 70.767-75.995 | 4.17 | 7.49 | -0.37 |
|  | *Qtss.saw-7B.1* | BLUP | 7B | *7B_531434068* | *7B_532468716* | *7B_455274386* | 117.259-124.687 | 3.74 | 5.58 | -0.20 |
|  | *Qtss.saw-7B.2* | 21HC | 7B | *7B_111991402* | *7B_292810098* | *7B_111991402* | 129.139-138.174 | 4.22 | 7.96 | -0.34 |
|  |  | 19YD | 7B | *7B_111991402* | *7B_292810098* | *7B_111991402* | 129.139-138.174 | 2.94 | 4.26 | -0.23 |
|  | *Qtss.saw-7B.3* | 20HC | 7B | *7B_678583143* | *7B_690048293* | *7B_678583143* | 59.192-65.465 | 3.19 | 6.22 | -0.33 |
| FSS | *Qfss.saw-2B.2* | 19YD | 2B | *2B_76515060* | *2B_76515060* | *2B_54768734* | 278.205-296.593 | 3.08 | 8.26 | 0.46 |
|  |  | 20YD | 2B | *2B_76515060* | *2B_76515060* | *2B_54768734* | 278.205-296.593 | 6.60 | 15.65 | 0.47 |
|  |  | 19YC | 2B | *2B_76515060* | *2B_76515060* | *2B_54768734* | 278.205-296.593 | 7.06 | 13.69 | 0.50 |
|  |  | 21YC | 2B | *2B_76515060* | *2B_76515060* | *2B_54768734* | 278.205-296.593 | 3.47 | 6.29 | 0.36 |
|  |  | 19HC | 2B | *2B_76515060* | *2B_76515060* | *2B_54768734* | 278.205-296.593 | 3.81 | 8.44 | 0.47 |
|  |  | BLUP | 2B | *2B_76515060* | *2B_76515060* | *2B_54768734* | 278.205-296.593 | 6.93 | 17.70 | 0.39 |
|  | *Qfss.saw-1A* | 21YD | 1A | *1A_567714120* | *1A_567714120* | *1A_568327780* | 0-1.46 | 3.25 | 6.22 | -0.34 |
|  | *Qfss.saw-2A* | 19HC | 2A | *2A_756911623* | *2A_756493154* | *2A_747084727* | 27.109-49.324 | 2.58 | 5.48 | -0.37 |
|  | *Qfss.saw-2B.3* | 19YC | 2B | *2B_774697389* | *2B_774697389* | *2B_737862890* | 96.742-119.008 | 2.76 | 6.07 | 0.35 |
|  |  | 19YC | 2B | *2B_774697389* | *2B_774697389* | *2B_737862890* | 96.742-119.008 | 2.76 | 6.07 | 0.35 |
|  | *Qfss.saw-2B.1* | 21YD | 2B | *2B_690211134* | *2B_712761198* | *2B_690211134* | 188.803-191.932 | 4.79 | 9.70 | 0.42 |
|  | *Qfss.saw-3B* | 20HC | 3B | *3B_586733548* | *3B_586733548* | *3B_592271369* | 39.632-42.79 | 3.01 | 6.44 | 0.34 |
|  | *Qfss.saw-3D* | 20YC | 3D | *3D_496504811* | *3D_494560750* | *3D_484624176* | 0-7.309 | 3.06 | 6.22 | -0.32 |
|  | *Qfss.saw-4A.1* | 19HC | 4A | *4A_17717246* | *4A_15121018* | *4A_17717246* | 0-3.357 | 3.01 | 6.50 | 0.40 |
|  | *Qfss.saw-4A.2* | 19HC | 4A | *4A_200909913* | *4A_444151741* | *4A_290138679* | 42.541-44.625 | 4.60 | 8.10 | 0.46 |
|  |  | 21HC | 4A | *4A_200909913* | *4A_444151741* | *4A_290138679* | 42.541-44.625 | 4.71 | 9.71 | 1.78 |
|  | *Qfss.saw-4A.3* | 21HC | 4A | *4A_222463171* | *4A_222463171* | *4A_133469615* | 58.176-59.218 | 2.87 | 6.23 | -1.13 |
|  |  | 21YD | 4A | *4A_133469615* | *4A_222463171* | *4A_133469615* | 58.176-59.218 | 4.09 | 8.73 | 0.41 |
|  | *Qfss.saw-4B* | 20YD | 4B | *4B_426604495* | *4B_426604495* | *4B_428975776* | 0-14.29 | 4.60 | 9.57 | 0.39 |
|  | *Qfss.saw-4D* | 19YC | 4D | *4D_370155010* | *4D_412935637* | *4D_370155010* | 21.321-23.941 | 4.12 | 6.77 | -0.35 |
|  | *Qfss.saw-5B.1* | 19YC | 5B | *5B_429637379* | *5B_421696566* | *5B_429637379* | 70.767-75.995 | 5.93 | 10.96 | -0.46 |
|  | *Qfss.saw-6A* | 21YC | 6A | *6A_539231010* | *6A_558886182* | *6A_535892032* | 29.932-37.786 | 5.58 | 10.79 | 1.06 |
|  | *Qfss.saw-7A.1* | 19HC | 7A | *7A_53005856* | *7A_47338564* | *7A_53005856* | 1.173-4.062 | 2.74 | 5.58 | 0.38 |
|  | *Qfss.saw-7A.2* | 20HC | 7A | *7A_617110161* | *7A_617110161* | *7A_632525808* | 94.31-95.933 | 7.87 | 15.90 | 1.09 |
|  | *Qfss.saw-7B.1* | 19HC | 7B | *7B_703502819* | *7B_703502819* | *7B_693240384* | 36.08-46.646 | 2.69 | 5.27 | -0.37 |
|  | *Qfss.saw-7B.2* | 20HC | 7B | *7B_678583143* | *7B_690048293* | *7B_678583143* | 59.192-65.465 | 3.02 | 6.94 | -0.35 |
| BSSS | *Qbsss.saw-2B.2* | 19YD | 2B | *2B_76515060* | *2B_76515060* | *2B_54768734* | 278.205-296.593 | 11.02 | 19.51 | 0.37 |
|  |  | 20YC | 2B | *2B_76515060* | *2B_76515060* | *2B_54768734* | 278.205-296.593 | 3.30 | 6.80 | 0.18 |
|  |  | 19HC | 2B | *2B_60980426* | *2B_76515060* | *2B_54768734* | 278.205-296.593 | 3.49 | 6.84 | 0.13 |
|  |  | 20HC | 2B | *2B_60980426* | *2B_76515060* | *2B_54768734* | 278.205-296.593 | 2.87 | 4.86 | 0.11 |
|  |  | BLUP | 2B | *2B_76515060* | *2B_76515060* | *2B_54768734* | 278.205-296.593 | 6.96 | 11.12 | 0.11 |
|  | *Qbsss.saw-5A.1* | 19HC | 5A | *5A_682703894* | *5A_682703894* | *5A_684699297* | 0-6.813 | 3.15 | 6.46 | -0.12 |
|  |  | 20HC | 5A | *5A_682703894* | *5A_682703894* | *5A_684699297* | 0-6.813 | 3.98 | 7.37 | -0.14 |
|  |  | 19YC | 5A | *5A_684699297* | *5A_682703894* | *5A_684699297* | 0-6.813 | 3.79 | 8.64 | -0.17 |
|  |  | BLUP | 5A | *5A_682703894* | *5A_682703894* | *5A_684699297* | 0-6.813 | 5.35 | 8.68 | -0.10 |
|  | *Qbsss.saw-2B.1* | 21YC | 2B | *2B_712761198* | *2B_712761198* | *2B_690211134* | 188.803-191.932 | 4.81 | 8.51 | 0.20 |
|  | *Qbsss.saw-3B* | 20YC | 3B | *3B_606834474* | *3B_640707875* | *3B_606834474* | 53.809-60.622 | 2.55 | 5.31 | 0.15 |
|  | *Qbsss.saw-3D* | BLUP | 3D | *3D_494560750* | *3D_494560750* | *3D_484624176* | 0-7.309 | 3.00 | 4.71 | 0.07 |
|  |  | 21YC | 3D | *3D_496504811* | *3D_494560750* | *3D_484624176* | 0-7.309 | 2.86 | 4.95 | 0.16 |
|  | *Qbsss.saw-4B.1* | 20YD | 4B | *4B_157290188* | *4B_126383632* | *4B_196858799* | 26.97-28.982 | 4.63 | 9.50 | -0.17 |
|  | *Qbsss.saw-4B.2* | 20YD | 4B | *4B_47457515* | *4B_42942201* | *4B_47457515* | 86.037-88.884 | 5.19 | 11.03 | -0.19 |
|  | *Qbsss.saw-4D.1* | 20HC | 4D | *4D_48697668* | *4D_15772687* | *4D_48697668* | 0-7.629 | 3.43 | 6.16 | 0.12 |
|  | *Qbsss.saw-4D.2* | 19YD | 4D | *4D_370155010* | *4D_412935637* | *4D_370155010* | 21.321-23.941 | 4.30 | 7.50 | -0.22 |
|  | *Qbsss.saw-5A.2* | 21YC | 5A | *5A_390751340* | *5A_394562478* | *5A_344530310* | 194.428-200.164 | 3.43 | 6.12 | -0.17 |
|  | *Qbsss.saw-5A.3* | BLUP | 5A | *5A_69243123* | *5A_83635094* | *5A_47142626* | 206.245-207.595 | 3.36 | 5.14 | -0.08 |
|  | *Qbsss.saw-5A.4* | 19HC | 5A | *5A_19238887* | *5A_19238887* | *5A_18501529* | 236-244.147 | 2.97 | 6.48 | -0.12 |
|  | *Qbsss.saw-5B* | 21HC | 5B | *5B_30521998* | *5B_30521998* | *5B_21188335* | 23.215-30.06 | 3.27 | 6.70 | 0.08 |
|  | *Qbsss.saw-6A* | 19YD | 6A | *6A_579445854* | *6A_579445854* | *6A_579841183* | 15.562-16.584 | 2.58 | 4.11 | -0.17 |
|  | *Qbsss.saw-6B.1* | 19YC | 6B | *6B_33068670* | *6B_33068670* | *6B_113657737* | 15.161-35.756 | 3.73 | 7.38 | 0.16 |
|  | *Qbsss.saw-6B.2* | 21HC | 6B | *6B_561258204* | *6B_561258204* | *6B_521185391* | 80.082-81.881 | 5.15 | 10.93 | 0.10 |
| TSSS | *Qtsss.saw-2B* | 19YC | 2B | *2B_718545059* | *2B_722682300* | *2B_718545059* | 20.105-21.948 | 3.12 | 6.07 | -0.22 |
|  | *Qtsss.saw-3A.1* | 21HC | 3A | *3A_741822204* | *3A_746964504* | *3A_741822204* | 0.521-14.41 | 2.58 | 5.30 | -0.06 |
|  | *Qtsss.saw-3A.2* | 20YC | 3A | *3A_730807220* | *3A_730807220* | *3A_732723387* | 54.522-61.558 | 2.82 | 5.50 | 0.18 |
|  | *Qtsss.saw-3A.3* | 21HC | 3A | *3A_724845751* | *3A_727373439* | *3A_719943969* | 79.627-101.963 | 4.19 | 8.80 | 0.08 |
|  | *Qtsss.saw-3A.4* | 19HC | 3A | *3A_638328149* | *3A_646670697* | *3A_638328149* | 184.913-186.476 | 2.75 | 5.98 | -0.09 |
|  | *Qtsss.saw-3B* | 19YD | 3B | *3B_115235989* | *3B_71119820* | *3B_115235989* | 0-0.522 | 3.08 | 9.51 | -0.29 |
|  | *Qtsss.saw-4D* | 20YD | 4D | *4D_15772687* | *4D_15772687* | *4D_48697668* | 0-7.629 | 2.91 | 6.75 | 0.10 |
|  | *Qtsss.saw-5B* | 21YC | 5B | *5B_654359522* | *5B_610798888* | *5B_654359522* | 275.112-310.501 | 2.56 | 5.34 | 0.24 |
|  |  | BLUP | 5B | *5B_654359522* | *5B_610798888* | *5B_654359522* | 275.112-310.501 | 3.45 | 7.16 | 0.09 |
|  | *Qtsss.saw-6B* | 21YD | 6B | *6B_521185391* | *6B_561258204* | *6B_521185391* | 80.082-81.881 | 2.84 | 5.64 | -0.15 |
|  |  | BLUP | 6B | *6B_470807066* | *6B_474153213* | *6B_470807097* | 85.983-87.548 | 5.16 | 9.28 | -0.10 |
|  | *Qtsss.saw-7B* | BLUP | 7B | *7B_725766535* | *7B_727844225* | *7B_725766535* | 0-4.685 | 4.84 | 8.68 | 0.10 |
| GNS | *Qgns.saw-5B.2* | 19YD | 5B | *5B_603868252* | *5B_610798888* | *5B_654359522* | 275.112-310.501 | 3.73 | 5.34 | -2.06 |
|  |  | 21YC | 5B | *5B_654359522* | *5B_610798888* | *5B_654359522* | 275.112-310.501 | 3.30 | 7.20 | -2.41 |
|  |  | BLUP | 5B | *5B_603868252* | *5B_610798888* | *5B_654359522* | 275.112-310.501 | 4.31 | 8.21 | -1.17 |
|  | *Qgns.saw-7A.1* | 19YD | 7A | *7A_658134960* | *7A_657918003* | *7A_658134960* | 112.675-113.208 | 2.78 | 4.55 | 1.79 |
|  |  | 19YC | 7A | *7A_657918003* | *7A_657918003* | *7A_675589691* | 112.675-113.208 | 2.83 | 5.42 | 1.86 |
|  |  | 20YC | 7A | *7A_657918003* | *7A_657918003* | *7A_675589691* | 112.675-136.68 | 3.25 | 6.80 | 1.99 |
|  |  | 21HC | 7A | *7A_657918003* | *7A_657918003* | *7A_675589691* | 112.675-136.68 | 5.19 | 9.94 | 2.13 |
|  |  | BLUP | 7A | *7A_658134960* | *7A_657918003* | *7A_658134960* | 112.675-113.208 | 4.06 | 9.44 | 1.20 |
|  | *Qgns.saw-4D* | 21YC | 4D | *4D_15772687* | *4D_15772687* | *4D_48697668* | 0-7.629 | 4.27 | 7.66 | -2.46 |
|  |  | 20YC | 4D | *4D_48697668* | *4D_15772687* | *4D_48697668* | 0-7.629 | 3.43 | 6.71 | -2.02 |
|  |  | 19HC | 4D | *4D_193777167* | *4D_15772687* | *4D_48697668* | 0-7.629 | 5.63 | 10.80 | -2.44 |
|  | *Qgns.saw-1A* | 21YD | 1A | *1A_567714120* | *1A_567714120* | *1A_568327780* | 0-1.46 | 3.72 | 6.60 | -2.03 |
|  |  | 21HC | 1A | *1A_568327780* | *1A_567714120* | *1A_568327780* | 0-1.46 | 5.79 | 11.16 | -0.92 |
|  |  | 21YC | 1A | *1A_568327780* | *1A_567714120* | *1A_568327780* | 0-1.46 | 2.56 | 4.47 | -2.25 |
|  |  | BLUP | 1A | *1A_567714120* | *1A_567714120* | *1A_568327780* | 0-1.46 | 2.75 | 5.25 | -1.88 |
|  | *Qgns.saw-4A* | 20YD | 4A | *4A_660040853* | *4A_661246903* | *4A_660040853* | 150.313-150.835 | 3.28 | 6.26 | -1.55 |
|  | *Qgns.saw-5B.1* | 20HC | 5B | *5B_21188335* | *5B_30521998* | *5B_21188335* | 23.215-30.06 | 4.90 | 10.14 | -2.47 |
|  | *Qgns.saw-6B* | 21YC | 6B | *6B_113657737* | *6B_33068670* | *6B_113657737* | 15.161-35.756 | 4.14 | 7.55 | 2.41 |
|  | *Qgns.saw-6A* | 21YD | 6A | *6A_579445807* | *6A_579445854* | *6A_579841183* | 15.562-16.584 | 6.45 | 11.87 | -4.04 |
|  |  | 20YD | 6A | *6A_579445854* | *6A_579445854* | *6A_579841183* | 15.562-16.584 | 4.07 | 7.76 | -1.67 |
|  | *Qgns.saw-2B.1* | 19YC | 2B | *2B_718545059* | *2B_722682300* | *2B_718545059* | 20.105-21.948 | 3.02 | 5.82 | 2.02 |
|  | *Qgns.saw-2B.2* | 20HC | 2B | *2B_51057383* | *2B_76515060* | *2B_60980426* | 278.205-290.292 | 2.64 | 5.76 | -1.82 |
|  | *Qgns.saw-3A* | 19YD | 3A | *3A_722932445* | *3A_724845751* | *3A_722932445* | 93.949-110.634 | 6.55 | 13.55 | -5.39 |
|  | *Qgns.saw-7A.2* | 21YC | 7A | *7A_619878262* | *7A_619878262* | *7A_628669633* | 99.115-99.636 | 2.53 | 4.18 | 1.84 |
|  | *Qgns.saw-4B.1* | 20YD | 4B | *4B_426604495* | *4B_426604495* | *4B_428975776* | 0-14.29 | 7.87 | 15.83 | 2.57 |
|  |  | 20YC | 4B | *4B_426604495* | *4B_426604495* | *4B_428975776* | 0-14.29 | 2.99 | 7.50 | -2.24 |
|  | *Qgns.saw-4B.2* | 21YD | 4B | *4B_621666683* | *4B_621666683* | *4B_639958335* | 52.821-61.281 | 2.84 | 4.84 | -1.95 |
|  | *Qgns.saw-7B* | 21HC | 7B | *7B_292810098* | *7B_292810098* | *7B_111991402* | 129.139-138.174 | 3.00 | 5.58 | -1.59 |
|  | *Qgns.saw-2A* | 19HC | 2A | *2A_747084727* | *2A_756493154* | *2A_747084727* | 27.109-49.324 | 4.42 | 8.27 | -2.19 |

**Table S4.** Additive effects of six TSS-related QTLs, two BSSS-related QTLs and four GNS-related QTLs.

| Traits | No. of favortable alleles | Mean | Difference^b^ | Sample Size |
| --- | --- | --- | --- | --- |
| TSS | 0 | 18.37±0.27a^a^ | 0 | 5 |
|  | 1 | 18.61±0.48ab | 0.25 | 17 |
|  | 2 | 18.98±0.54ab | 0.61 | 32 |
|  | 3 | 19.37±0.67b | 1.00 | 57 |
|  | 4 | 19.48±0.59b | 1.11 | 49 |
|  | 5 | 20.54±0.89c | 2.17 | 25 |
|  | 6 | 20.61±0.32c | 2.25 | 7 |
| BSSS | 0 | 1.02±0.30a | 0 | 45 |
|  | 1 | 1.19±0.28b | 0.17 | 101 |
|  | 2 | 1.44±0.28c | 0.42 | 46 |
| GNS | 0 | 41.84±6.92a | 0 | 7 |
|  | 1 | 43.35±3.50ab | 1.51 | 72 |
|  | 2 | 45.44±3.30bc | 3.60 | 66 |
|  | 3 | 47.22±2.72cd | 5.38 | 35 |
|  | 4 | 48.25±3.89d | 6.41 | 12 |

^a^Values followed by the same letter are not significantly different at *p* = 0.05.

^b^Differences calculated using the entries with the postive alleles minus the entries with negative alleles.

**Table S5:** Additive effects of the QTL on *Qtss.saw-2B.1*, *Qtss.saw-2B.2* and *Qtss.saw-3B* for TSS in JN population.

| *Qtss.saw-2B.1* | *Qtss.saw-2B.2* | *Qtss.saw-3B* | Sample Size | TSS | Differencec | Increased percent (%)d |
| --- | --- | --- | --- | --- | --- | --- |
| *+*a | *+* | *+* | 39 | 20.17±0.61db | 1.38 | 7.33 |
| *+* | *+* | *-* | 22 | 19.70±1.17c | 0.9 | 4.81 |
| *-* | *+* | *+* | 15 | 19.50±0.64bc | 0.7 | 3.73 |
| *+* | *-* | *+* | 20 | 19.28±0.73abc | 0.48 | 2.56 |
| *-* | *+* | *-* | 14 | 19.42±1.02bc | 0.62 | 3.3 |
| *-* | *-* | *+* | 20 | 19.26±0.63abc | 0.46 | 2.45 |
| *+* | *-* | *-* | 30 | 19.16±0.56ab | 0.36 | 1.92 |
| *-* | *-* | *-* | 32 | 18.80±0.49a | 0 | 0 |

^a^ Plus and minus represent lines with and without the positive alleles of the target quantitative trait loci (QTL) based on the flanking markers and the corresponding QTL.

^b^ All pair means were compared using the Scheffe method. Values followed by the same lower case letter are not significantly different at *p* 0.05.

^c^ Differences calculated using the entries with the three positive alleles minus the entries with three negative alleles

^d^ Increased percent (%) is that differences is divided by the entries with three negative alleles and then multiplied 100

**Table S6:**  Functional annotation and enrichment of TSS QTL on chromosome 2BL.

**Table S7:** Functional annotation and enrichment of TSS QTL on chromosome 3B.

**Supplementary Figures**

**Figure. S1** Expression profile of candidate genes of *Qtss.saw-2B.1*

**Figure. S2** Expression profile of candidate genes of *Qtss.saw-3B*
